# Optimization of Touchscreen-Based Behavioral Paradigms in Mice: Implications for Building a Battery of Tasks Taxing Learning and Memory Functions

**DOI:** 10.1371/journal.pone.0100817

**Published:** 2014-06-24

**Authors:** David Delotterie, Chantal Mathis, Jean-Christophe Cassel, Cornelia Dorner-Ciossek, Anelise Marti

**Affiliations:** 1 Laboratoire de Neurosciences Cognitives et Adaptatives, UMR 7364, Université de Strasbourg-CNRS, Faculté de Psychologie, Strasbourg, France; 2 Boehringer Ingelheim Pharma GmbH & Co KG, Dept. of CNS Diseases Research, Biberach an der Riss, Germany; Inserm U837, France

## Abstract

Although many clinical pathological states are now detectable using imaging and biochemical analyses, neuropsychological tests are often considered as valuable complementary approaches to confirm diagnosis, especially for disorders like Alzheimer’s or Parkinson’s disease, and schizophrenia. The touchscreen-based automated test battery, which was introduced two decades ago in humans to assess cognitive functions, has recently been successfully back-translated in monkeys and rodents. We focused on optimizing the protocol of three distinct behavioral paradigms in mice: two variants of the Paired Associates Learning (PAL) and the Visuo-Motor Conditional Learning (VMCL) tasks. Acquisition of these tasks was assessed in naive *versus* pre-trained mice. In naive mice, we managed to define testing conditions allowing significant improvements of learning performances over time in the three aforementioned tasks. In pre-trained mice, we observed differential acquisition rates after specific task combinations. Particularly, we identified that animals previously trained in the VMCL paradigm subsequently poorly learned the sPAL rule. Together with previous findings, these data confirm the feasibility of using such behavioral assays to evaluate the power of different models of cognitive dysfunction in mice. They also highlight the risk of interactions between tasks when rodents are run through a battery of different cognitive touchscreen paradigms.

## Introduction

Neuropsychological tests historically represent valuable tools to diagnose and follow up neurodegenerative and psychiatric disorders. In many instances, they allow a precise discrimination between close pathological states affecting cognition [1]. Numerous cognitive domains can thereby be tested, among which executive functions, attention, different aspects of short- and long-term memories, etc. One of the best examples illustrating the importance of such tools is probably the mini mental state examination (MMSE). This composite cognitive test was introduced in 1975 in the field of clinical research to detect possible cognitive impairments/probable dementia in aged patients [2]–[4]. Almost forty years later, despite the increasing use of biomarkers for detection of Alzheimer’s disease [5], [6], it is worth noticing that this readout is still recommended for the evaluation of demented patients [7] or the recruitment of patients with mild to moderate dementia for clinical trials [8]–[11].

If various tests have been progressively implemented over the last decades, the Cambridge neuropsychological test automated battery (CANTAB) certainly deserves a particular attention due to its translational dimension [12]. Initially established in humans, neuropsychological tests included in this computerized battery are based on a universal principle: subjects have to respond to variously-shaped stimuli displayed on a sensitive touchscreen according to a defined rule. These tasks present the great advantage to be directly translatable from humans to non-human primates after no or sometimes only minor adaptations [13]. Interestingly, in macaques infected with the simian immunodeficiency virus (SIV) neuropsychological deficits appear similar to those described in human AIDS patients [14], and the same keeps true in aged rhesus monkeys compared to healthy aged humans [15]. Moreover, the assessment of cognitive abilities through this methodology is sensitive to drug manipulations in both monkeys and humans [16]–[19].

Recent reports have emphasized the need for more translational preclinical assays in animal models to better predict the efficacy of putative therapeutic agents in clinical studies [20]–[23]. Capitalizing on the additional value of new emerging models based on advances in transgenesis techniques [24], [25], Bussey and collaborators gradually introduced the touchscreen-automated testing method in rats and mice [26]–[28]. As in humans, rodents are expected to respond to visual stimuli displayed on a touchscreen according to a specific rule. Nevertheless, each associative learning of a given cognitive task requires extensive training. Correct nose pokes are thus rewarded with an appetitive reinforcer in food-deprived animals, which contributes to strengthen motivation and to decrease the stress component. Various behavioral touchscreen-based tasks pertaining to different cognitive functions and presenting the added benefit of automated measures in a controlled environment have thus been adapted in rodents [29]–[31]. Furthermore, several articles argue in favor of their use to screen or validate the predictivity of new animal models, especially with regard to schizophrenia and Alzheimer’s disease [32]–[37].

The goals of the present work were to optimize in mice two cognitive touchscreen-based tasks, the paired-associates learning (PAL) and the visuo-motor conditional learning (VMCL) tasks and to validate whether they could be combined to evaluate the successive performances of animals tested in a battery of assays [38]–[40]. These paradigms have been studied in rats and are thought to depend on distinct brain structures, namely the hippocampus and the dorsal striatum, respectively [41]–[44]. Therefore, they could be, for instance, of high interest for the sequential cognitive evaluation of animal models of Alzheimer’s disease, which are generally impaired in hippocampal-dependent tasks [45], [46] but display preserved abilities in striatal-dependent procedural forms of learning [47]. So far, however, very few data are available in mice [48], [49]. We first explored (experiment A) the acquisition of two versions of the PAL task using similar (sPAL) or different (dPAL) stimuli to examine in that spatial paradigm the role of the nature of presented objects [44]. In order to optimize the VMCL task (experiment B), we then investigated the impact of various training conditions that had been previously identified as critical factors for subsequent acquisition (with or without “pretraining”; different limited holding times to respond to the screen; data not shown, obtained in pilot studies). Finally, we determined (experiment C) whether the acquisition of a first rule affected the way mice learned a second rule in another task.

## Results

### Experiment a: dPAL *vs* sPAL Tasks in Naive Animals

Three mice (one from the dPAL group, two from the sPAL group) out of the 16 naive animals were excluded from data analysis because they displayed no evidence for learning after 50 testing sessions (accuracy<60%).

**Figure 1:**
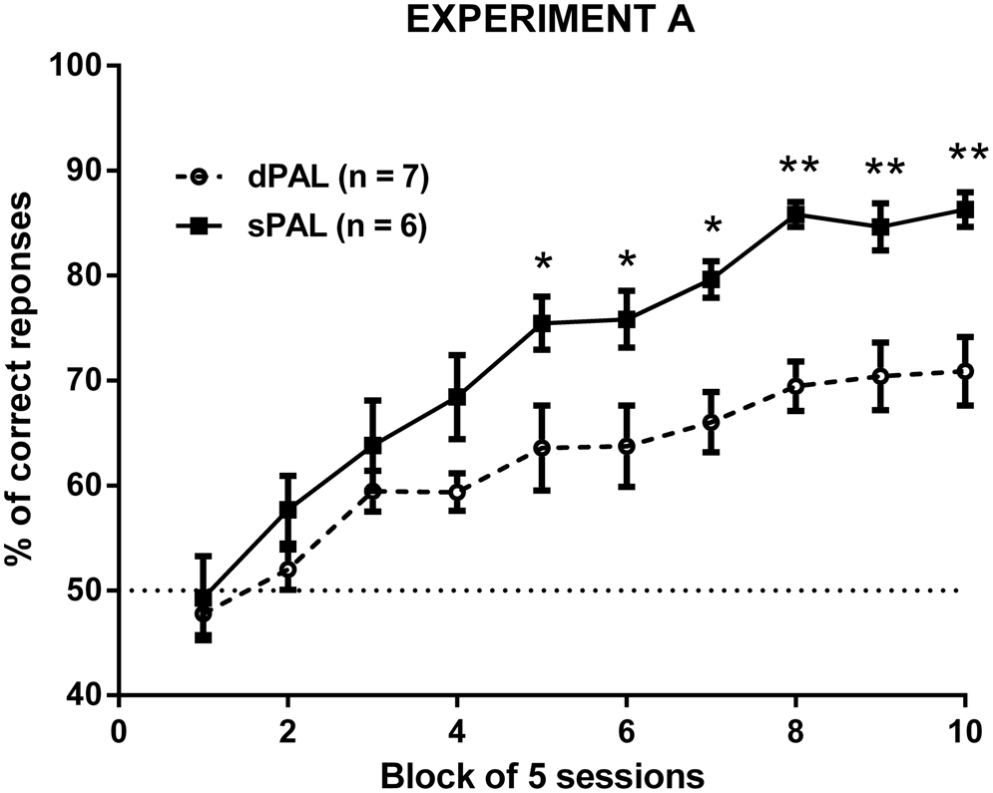
Acquisition curves in naive mice trained either in the sPAL (similar objects) or the dPAL (different objects) tasks. * p<0.05 and **p<0.01 vs the dPAL group.

To determine if performance changed over time, we plotted accuracy data in 10 blocks of 5 sessions for the 2 variants of the PAL task (see figure 1). There was a significant effect of Time (F(9,99) = 35.26; p<0.0001) and Task (F(1,11) = 17.18; p<0.01) when looking at the accuracy parameter. A significant Time × Task interaction was also found (F(9,99) = 2.19; p<0.05). *Post-hoc* analysis revealed that the difference between the 2 variants of the PAL task appeared from 25 sessions onwards (block 5; t(11) = 2.893; p<0.05) and even became more important after 40 testing sessions (block 8; t(11) = 3.988; p<0.01), suggesting an easier acquisition of the sPAL task.

In agreement with this observation, planned comparisons against the group trained in the sPAL task showed a significant higher number of correction trials in animals recorded in the dPAL task (see table 1; t(11) = 2.743; p<0.05). Interestingly, there were no significant differences between dPAL and sPAL groups regarding correct touch (t(11) = 0.411; p>0.05), incorrect touch (t(11) = 0.417; p>0.05) and magazine (t(11) = 0.508; p>0.05) latencies.

**Table 1:** Additional parameters measured in touchscreen boxes during experiments A, B and C.

Experiment	Task	Group	N	N° of CT	CTL (s)	ITL (s)	ML (s)
A	1	dPAL	7	26.91±1.92*	2.05±0.18	2.07±0.15	1.26±0.05
A	1	sPAL	6	20.03±1.51	1.96±0.10	2.14±0.09	1.22±0.05
B	1	VMCL - Conditions 1	8	11.18±0.72	0.79±0.07	0.35±0.04##	1.10±0.03
B	1	VMCL - Conditions 2	7	9.52±1.00	0.77±0.07	0.25±0.04	1.11±0.04
B	1	VMCL - Conditions 3	8	12.21±0.99	0.75±0.07	0.29±0.03	1.04±0.04
B	1	VMCL - Conditions 4	7	9.28±1.46	0.58±0.04	0.16±0.02	1.16±0.03
C	2	dPAL (after VMCL)	6	27.92±1.97	2.17±0.28	2.26±0.25	1.32±0.05
C	2	sPAL (after VMCL)	7	32.43±2.49**	2.95±0.54	2.83±0.44	1.50±0.11
C	2	VMCL (after dPAL)	7	11.10±1.11	1.07±0.26	0.46±0.13	1.16±0.07
C	2	VMCL (after sPAL)	7	10.85±0.73	0.78±0.10	0.30±0.04	1.16±0.07

CT: Correction Trials; CTL: Correct Touch Latency; ITL: Incorrect Touch Latency; ML: Magazine Latency.

All parameters are expressed as mean values ± SEM.

*p<0.05,

**p<0.01 *vs* sPAL group (task 1);

## p<0.01 *vs* VMCL conditions 4 group.

### Experiment B: VMCL Task in Naive Animals

Four different groups of 8 mice each were assessed in experiment B. Only two mice were excluded due to weak learning performance after 30 testing sessions (accuracy<75%). Consequently, groups 2 and 4 included only 7 mice.

**Figure 2:**
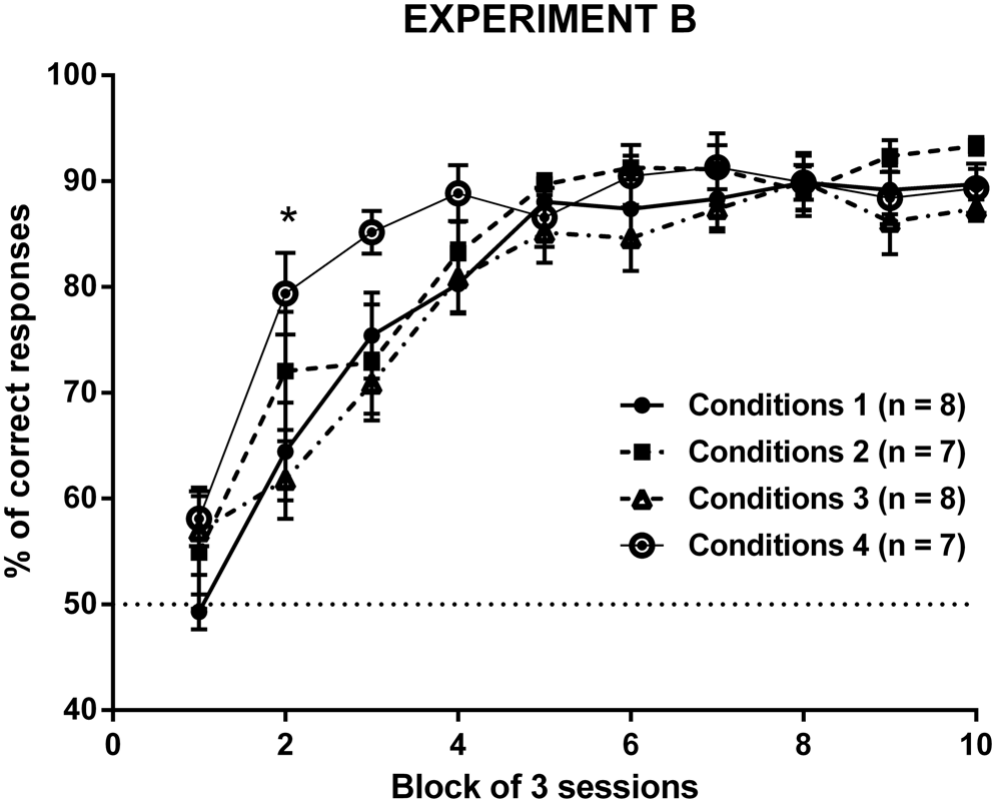
Acquisition curves in naive mice trained under various conditions in the VMCL task. *p<0.05 group 4 vs group 1 and p<0.01 group 4 vs group 3.

Accuracy data were plotted in 10 blocks of 3 sessions for all groups trained in the VMCL task (see figure 2). Analysis of accuracy showed a significant effect of Time (F(9,234) = 70.60; p<0.0001) and Training condition (F(3,26) = 3.00; p<0.05), but no interaction between the two factors in the VMCL task (F(27,234) = 1.37; ns). Additional *post-hoc* analyses indicated a difference between groups 1 and 4 and groups 3 and 4 only during the earliest learning sessions (block 2: t(13) = 3.480 and t(13) = 4.106; p<0.05 and p<0.01, respectively).

Such subtle learning changes in group 4 did not affect most of the global measures reported in table 1: no significant differences were observed when comparing groups 1 to 4 on the number of correction trials (F(3,29) = 1.769; p>0.05), correct touch (F(3,29) = 2.282; p>0.05) and magazine latencies (F(3,29) = 1.885; p>0.05). Nevertheless, we found a significant effect of Training condition on incorrect touch latency (F(3,29) = 6.293; p<0.01): mice trained in conditions 1 presented a significantly higher incorrect touch latency than those trained in conditions 4 (t(13) = 4.274, p<0.01).

### Experiment C: dPAL, sPAL and VMCL in Animals Previously Assessed in Touchscreen Tasks

**Figure 3:**
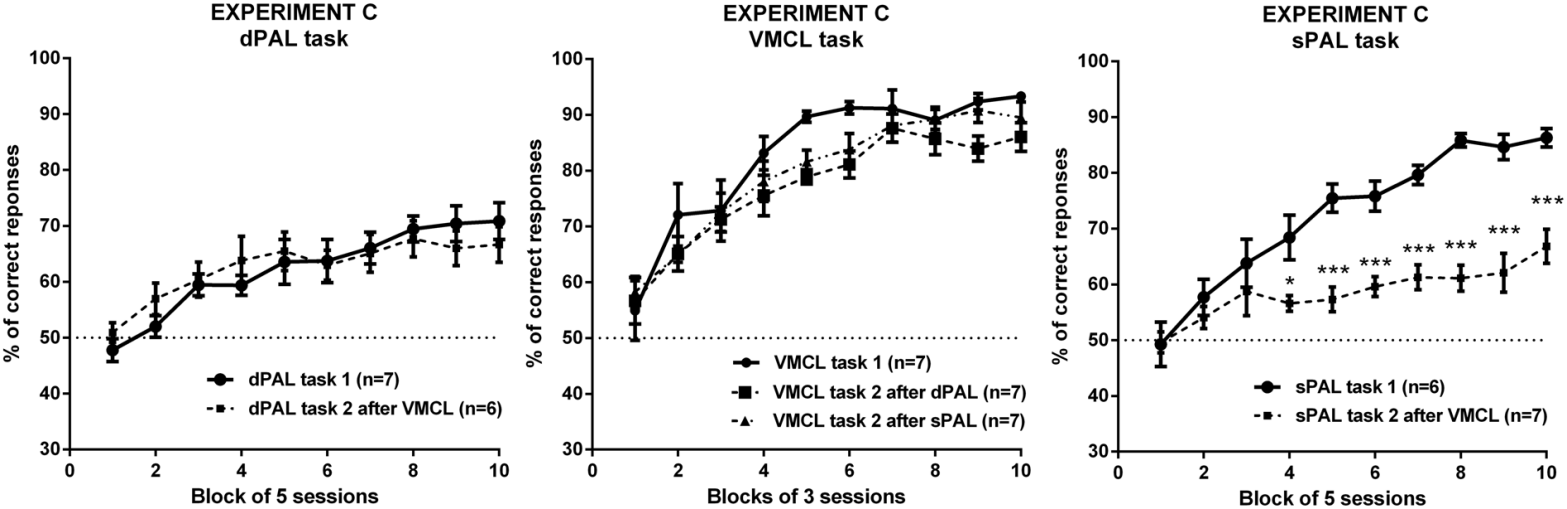
Effect of a previous training experience in touchscreen boxes on the acquisition of a new task. Comparison of the acquisition curves of naive vs trained mice in the dPAL (left panel), the VMCL (central panel) or the sPAL task (right panel). *p<0.05 and ***p<0.001 vs the sPAL (task 1).

Animals from experiment A (dPAL or sPAL) were assessed in the VMCL task, whereas animals from experiment B were assessed in 1 of the 2 variants of the PAL task. All groups were initially composed of 8 mice, but the same aforementioned criteria of accuracy were used to determine the final size of each group in experiment C: n = 6 mice in the dPAL task after the VMCL task; n = 7 mice in the sPAL task after the VMCL task; n = 7 mice trained in the VMCL task after the dPAL task; n = 7 mice trained in the VMCL task after the sPAL task. Accuracy data were plotted as described in experiments A and B (see figure 3); global measures were comparable to those shown in table 1.

There was a main effect of Time (F(9,99) = 19.02; p<0.0001) on accuracy in the dPAL task (figure 3, left panel), but no effect of Task experience (F(1,11) = 0.01; p>0.05) and no interaction between the two factors (F(9,99) = 1.28; p>0.05). Moreover, no significant difference was found among the different global measures (table 1) within tested groups: number of correction trials (t(11) = 0.367; p>0.05), correct touch (t(11) = 0.372; p>0.05), incorrect touch (t(11) = 0.679; p>0.05), and magazine (t(11) = 0.902; p>0.05) latencies.

Likewise, analysis of the accuracy in the VMCL task (figure 3, central panel) revealed a significant effect of Time (F(9,162) = 52.97; p<0.0001), but no effect of Task experience (F(2,18) = 3.16; p>0.05) and no interaction between the two factors (F(18,162) = 0.80; p>0.05). Furthermore, none of the additional measures summarized in table 1 was significantly different within tested groups: number of correction trials (F(2,18) = 0.783; p>0.05), correct touch (F(2,18) = 1.089; p>0.05), incorrect touch (F(2,18) = 1.860; p>0.05), and magazine (F(2,18) = 0.195; p>0.05) latencies. Altogether, these results suggest that an experience in a VMCL touchscreen task does not influence subsequent acquisition of a dPAL touchscreen task, and both versions of the PAL task do not influence the subsequent acquisition of the VMCL task.

However, unlike the 2 other tasks, analysis of the accuracy in the sPAL task (figure 3, right panel) indicated significant effects of Time (F(9,99) = 20.68; p<0.0001), Task experience (F(1,11) = 71.13; p<0.0001; from block 4, p<0.05) and an interaction between these factors (F(9,99) = 5.19; p<0.0001). Mice previously trained in the VMCL task were slower to acquire the sPAL task than naive mice. Importantly, this significant effect was accompanied by a significant increase of the number of correction trials (table 1; t(11) = 4.079; p<0.01), as well as a non-significant increase of both correct touch (t(11) = 1.677; p>0.05), incorrect touch (t(11) = 1.439; p>0.05), and magazine (t(11) = 2.135; p>0.05) latencies.

### Preference for a Specific Stimulus or Location in Touchscreen Tasks

**Figure 4:**
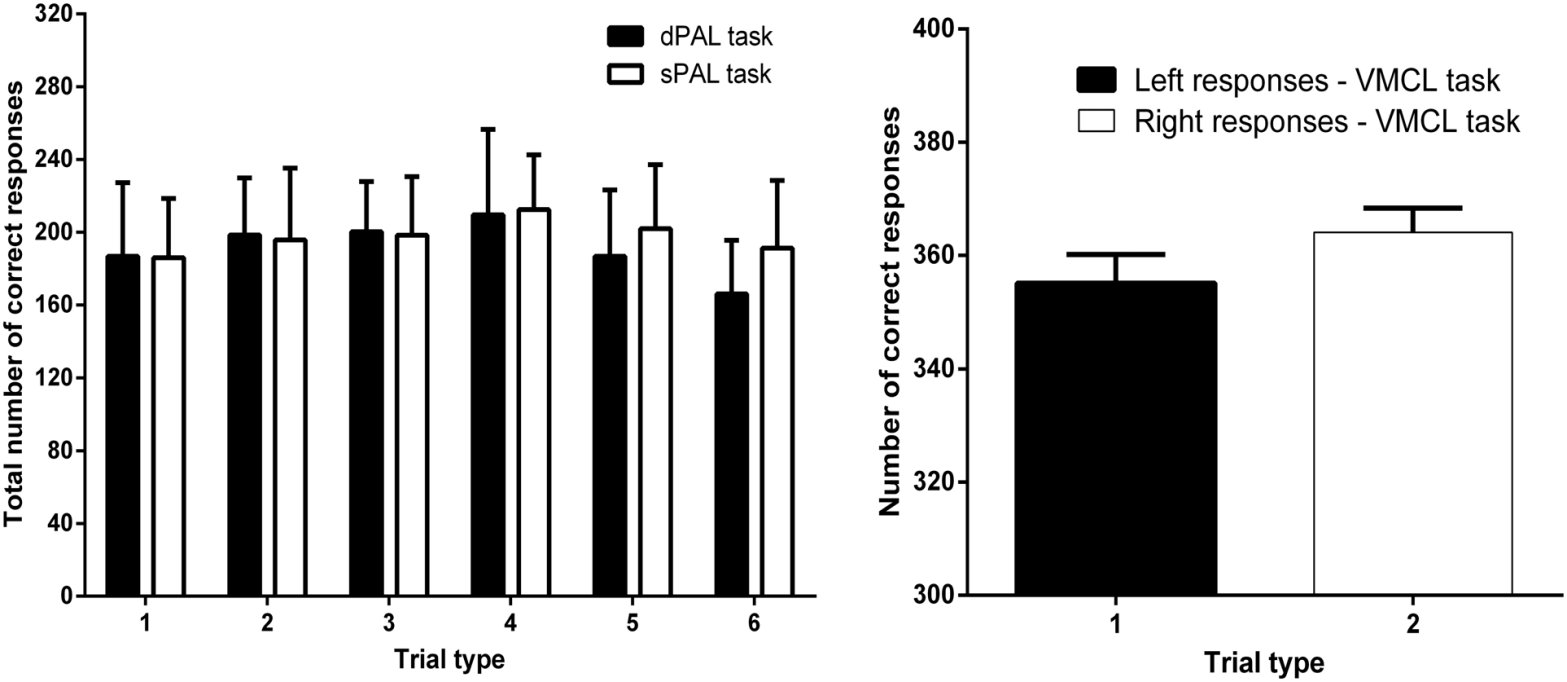
Global repartition of the correct responses of all animals assessed in the PAL tasks (left panel) or in the VMCL task (right panel). In both PAL paradigms, there are six possible object-place combinations. In the VMCL task, mice can only respond to the left or the right part of the screen after the first central nose-poke.

To explore the possibility of a preference for a specific configuration of stimuli among the different trial types, we analyzed for both variants of the PAL task the repartition of the total number of correct responses recorded in experiments A and C, independently of previous touchscreen experience. A similar calculation was made with regard to the VMCL task to determine whether mice presented a preference for a certain location (left *vs* right) from experiments B and C. Corresponding results are illustrated in figure 4.

In the dPAL task, we initially found a significant effect of the Trial type (F(5,66) = 3.050; p<0.05). A complementary *post-hoc* analysis showed that animals responded significantly more when trial types 3 or 4 were presented on the screen as compared to trial type 6 (respectively, t(22) = 3.189; p<0.05 et t(22) = 3.416; p<0.05). However, a close inspection of performance showed that this effect was mainly due to 2 mice which specifically occulted that trial type. After their exclusion (figure 4, left panel), significance vanished (F(5,54) = 1.760; p>0.05). In parallel, a similar analysis led for the sPAL task with raw effectives did not reveal any effect of the Trial type (F(5,66) = 0.830; p>0.05). These results suggest that mice acquiring one of the two variants of the PAL task do not learn the rule by partially using some of the displayed stimuli, but rather consider all combinations of visual stimuli to progressively define the nature of the rule.

In the VMCL task, there was no significant effect of the Trial type (t(86) = 1.344; p>0.05). This result is in agreement with the balanced expression of left *vs* right correct responses observed in these mice (figure 4, right panel) and confirms the absence of side preference in this task.

## Discussion

A first goal of this study was to validate testing conditions demonstrating the acquisition of three distinct cognitive, touchscreen-based tasks. Two variants of the paired-associates learning (PAL) task and one of the visuo-motor conditional learning (VMCL) task were used in mice. If instrumental touchscreen tasks present many advantages, as multiple cognitive domains can be assessed [50], their development remains quite challenging due to the numerous parameters that must be considered and adjusted. Critical factors such as the duration of the inter-trial interval (ITI), the number of trials per session, the nature and size of the stimuli can deeply influence performance [27], [28]. However, tasks presented in this paper have been adapted on the basis of previous works in rats [26], [44] and mice [48], facilitating their optimization in mice. Mice were able to perform all tasks with a significant improvement over time. Nose-pokes given to the correct or incorrect stimuli occurred quickly (always <3 s), indicating good reaction times and short decision-making. Moreover, low magazine latencies (around 1 s) demonstrated intact motivation towards a liquid reward (diluted condensed milk solution). Finally, it could be argued that if naive/previously trained mice were apparently able to learn the 3 different tasks, they might have developed preferences for stimuli or locations over time. However, there was no preference for stimuli or locations in our different experiments.

In experiment A, naive animals were trained in one if the two PAL variants (sPAL or dPAL tasks). Over extensive training, mice were able to identify the different stimuli (Flower, Plane, and Spider), to distinguish between locations (Left, Central, and Right) and to remember in which specific location each stimulus was systematically rewarded. The cognitive demand was expected to be lower in the sPAL task than in the dPAL task, as similar stimuli were presented within a same trial in that case. Indeed, the former task was acquired faster than the latter in rats [44], although the sPAL and dPAL learning curves merged at 85% of correct responses after 45 sessions. In our experiments, we observed similar initial patterns of learning, with the sPAL task being acquired quicker than the dPAL task, but unlike rats, dPAL and sPAL learning curves still diverged after 50 sessions of training (70% *vs* 85% of correct responses, respectively). The final performance of our mice trained in the dPAL task was also in accordance with a previous study in which mice tested in the same paradigm reached 80% correct responses after 95 testing sessions [48].

With regards to the VMCL task, for the first time we successfully transposed the task from rats [26] to mice. In a first experiment (data not shown), we had measured the ability of young naive male C57BL/6 mice to learn the VMCL rule using a rat paradigm with a few differences on the nature of the reward (pellets), the type of boxes (Med Associates boxes) and the characteristics of the ITI (a variable ITI of 40±30 s, which means a random value between 10 and 70 s). In these conditions, mice trained in similar pokey training stages reached 70% correct after 30 sessions in the VMCL task, which was in conflict with the quick acquisition of the task in rats [51]. We suspected the value of the ITI to be the determining factor and therefore decided to reduce its duration to 20 s as a fixed interval for both “pretraining” and VMCL tasks in experiment B. Other parameters were also adjusted as all naive mice were trained in touchscreen boxes using condensed milk as the reward. Groups 1 and 2 were recorded to evaluate the impact of a “pretraining” phase. Because the quick disappearance of the stimuli could also incite mice to approach the touchscreen and nose-poke more efficiently the stimuli, we also measured whether mice trained with a limited holding time during both “pretraining” and VMCL task (5 s for group 3; 3 s for group 4) would learn easier the VMCL task. Surprisingly, all groups quickly learned the VMCL task under these conditions, achieving 90% of correct responses after 15–18 sessions of 30 trials. This acquisition rate was almost comparable to the performance of rats in the VMCL task (90% of correct responses after 6 sessions of 100 trials), although rats had been trained with more difficult conditions, especially the disappearance of the discriminative central stimulus which increases the mnesic component of the task during each choice phase [51].

The use of a battery of cognitive touchscreen tasks using similar stimuli, responses and outcomes has been recently highlighted and emphasized [50], [52]. Therefore, we decided to investigate to which extent a first assessment in a touchscreen task would influence the acquisition of a second task differing by the nature of its rule. We noticed no difference between acquisition of the VMCL or the dPAL tasks between naive or trained mice, but observed an interesting gap in the sPAL task: whilst animals first trained in the sPAL task normally acquired the VMCL task, those first trained in the VMCL task displayed a learning deficit in the sPAL task, reaching only 65% of correct responses after a total of 50 sessions. These results suggest that under certain circumstances, one form of learning could interfere with the subsequent acquisition of a second, harder task. They also underline the putative involvement of common neural substrates in the sPAL and VMCL tasks. Observing that intra-hippocampal infusions of drugs had no effect on post-acquisition performance of the sPAL task in rats [44], Talpos hypothesized that similarly to the VMCL task, the sPAL task could be solved via a conditional rule of the type “If stimulus A appears, then choose location 1; if stimulus B appears, then choose location 2; if stimulus C appears, then choose location 3″. We globally agree with that view. Final performance of naive mice trained in the sPAL task converged towards those of naive mice trained in the VMCL task (85–90% of correct responses), albeit the acquisition process required a higher number of sessions in the first case, probably due to task difficulty. Additionally, if we admit that animals base their responses on conditional rules to achieve both VMCL and sPAL tasks, it makes sense noticing that mice first trained in the most difficult task (sPAL task) can efficiently learn a simpler rule (VMCL task), whereas mice first trained in the easiest task (VMCL task) struggle to learn a harder rule (sPAL task).

In parallel, our results also confirm the possibility to measure dPAL and VMCL performances in mice and to combine these tasks to evaluate cognitive impairment. In particular, it should be of great interest to investigate the effects of hippocampal (HPC) *vs* dorso-striatal (DS) lesions on the acquisition of both tasks. Indeed, compelling evidence indicate that the dPAL task primarily depends upon the hippocampal integrity. First, the human version of the task [12], [53] allows measuring direct episodic memory performance and detecting early impairments in patients with mild cognitive impairment (MCI), Alzheimer’s disease [54]–[56] or schizophrenia [57], [58]. Moreover, using fMRI, it has recently been shown that the low performance of MCI patients in the dPAL task specifically coincided with a lower hippocampal activation when the task demand was increased [59]. Second, although the rodent version of the dPAL task assesses object-in-place memory rather than episodic memory – with object-place (what-where) associations being gradually encoded during training –, the hippocampus plays an important role during retention of this type of information. Indeed, post-acquisition, intra-hippocampal infusion of MK-801, lidocaine or CNQX produces a significant decrease of dPAL performance in rats [44]. Third, data from other paired-associates learning tasks support a hippocampal implication during the acquisition of the task in rats, especially when one of the two dimensions to associate is a spatial feature [60]–[62].

By contrast, the VMCL task has historically been introduced as a stimulus-response learning task in rats thirty years ago [63]. In this construct, animals had initially to learn a conditional rule of the type “If lights are flashing FAST, press the right lever; if lights are flashing SLOW, press the left one”. Later this procedure was replaced by another conditional rule of the type “If stimulus A appears, then go left; if stimulus B appears, then go right” along with the emergence of the touchscreen method [26]. As expected in such a habitual task, control rats quickly learned the rule and reached a plateau performance (about 90% correct responses) after only a few sessions. Different studies based on excitotoxic lesions have shown the involvement of a corticostriatal network relying on intact dorso-lateral striatum [41], [42] and cingulate cortex [26] in this task. Accordingly, animals’ acquisition and subsequent performance were spared after lesions of the hippocampus [43], the prelimbic cortex, thalamic nuclei [64], perirhinal and postrhinal cortices or after a fornix transection [51]. Given that a similar rule resulted in fast acquisition curves in our mice, the task is most likely linked to the integrity of the same brain regions than in rats.

## Conclusion

Optimizing training conditions in translational paradigms is an important step as mice represent an increasingly used species in preclinical research, notably since the emergence of genetic models. Here, we demonstrate that, like rats, normal mice can successfully learn three appetitive touchscreen rules defining associations between objects and locations: the dPAL task, the sPAL task and the VMCL task. Using the touchscreen method, reliable parameters make it possible to monitor the animals’ performance in the absence of object/location preference and to check their motivational state throughout the experiment. We also show that although it may be appropriate to use the dPAL task and the VMCL task in a cognitive testing battery, as numerous papers underpin the involvement of distinct neural substrates in similar tasks, the cumulative assessment of mice in both the sPAL and VMCL task appears to be more risky. Future studies should now examine the effects of hippocampal *vs* dorso-striatal lesions in mice trained in the dPAL or the VMCL tasks.

## Materials & Methods

### Ethics Statement

All protocols included in this study and procedures related to Animal Care and Treatment were conducted with the specific approval of the appropriate governmental agency (Regierungspräsidium Tübingen, Germany) and performed in an AAALAC (Association for Assessment and Accreditation of Laboratory Animal Care International)-accredited facility in accordance with European Union guidelines (European Community Council Directive 2010/63/UE). All efforts were made to minimize animal suffering.

### Animals

48 male C57BL/6JRj mice were obtained from Janvier (France). They were 8–10 weeks old (23–27 g) at the start of food deprivation. Upon their arrival, mice were placed in a temperature- and humidity-controlled environment under a 12 h light/dark cycle (lights on 06∶00 h). They were individually housed in plastic cages (dimensions: length = 26 cm; width = 21 cm; height = 14 cm) to allow a more accurate follow-up of their daily food-intake. Each cage contained wood shaving bedding, and a red transparent plastic nest box and paper strips to provide some environmental enrichment. Animals were first given a week of habituation to the environmental conditions of our animal facility. Meanwhile, mice were weighed three times to determine their respective basal free-feeding body weight. The body weight was then slowly reduced and maintained at 85–90% of its free-feeding value throughout behavioral testing. Behavioral assessments were conducted during the light phase of the light/dark cycle. Mice were trained 5–6 days/week and rewarded in touchscreen devices with a liquid reward (condensed milk, Milch Mädchen, Nestlé, Germany; half diluted in water). They were directly weighed and fed upon return to the home cage after each daily session. Water was available *ad libitum*.

### Apparatus

The touchscreen-based apparatus consisted in an operant chamber housed within a sound and light attenuating box. Every trapezoidal-shaped chamber (respective dimensions: big basis = 25 cm; small basis = 6 cm; height = 18 cm) was individually equipped with a house light and a tone generator, and had been especially designed to focus the attention of the animal towards the touchscreen placed at one end of the chamber (model #80614, Bussey Mouse Touchscreen Chamber, Campden Instruments, U.K.). The liquid reward dispenser delivering condensed milk into a magazine was located at the opposite end of the chamber. The touchscreen was permanently covered by a black Plexiglas 3-holes mask. Three square windows (side dimensions: length = 7 cm; height = 7 cm) were separated by 0.4 cm and located at a height of 3.6 cm from the floor of the chamber. Through these windows, different visual stimuli could be shown on the screen (max. 1 stimulus per window). Stimulus presentation and reward delivery timing were both controlled by a graphical task design software (ABET II Touch software, model #89505, Campden Instruments, U.K.) according to the automated detection of animal nose-pokes specifically oriented towards the screen and the magazine.

### Behavioral Procedures

#### Experiment A: dPAL vs sPAL tasks in naive animals

**Figure 5:**
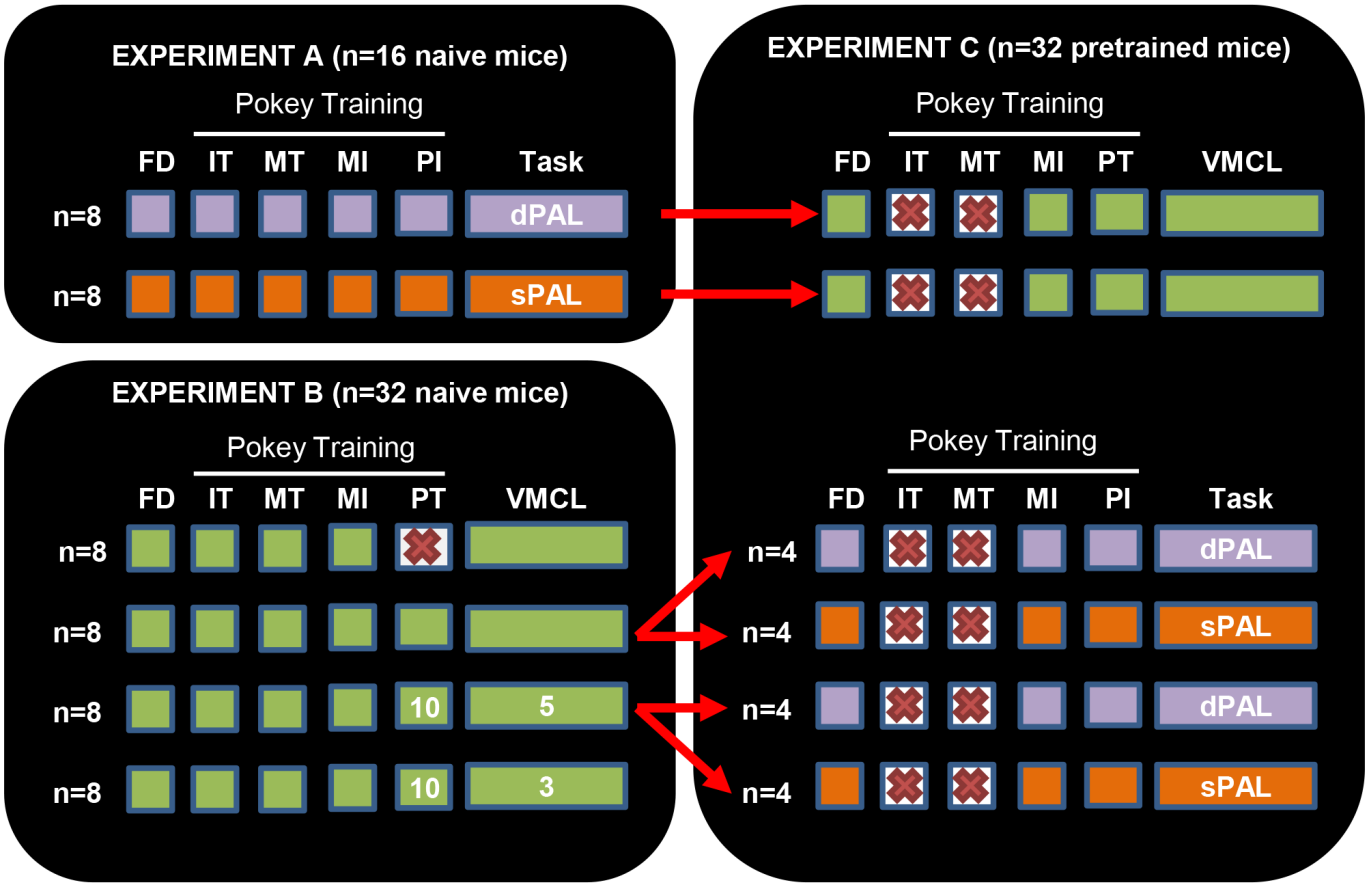
Global design of touchscreen experiments. In experiments A and B, naive mice were trained in the dPAL, sPAL or VMCL tasks according to specific learning conditions (groups 1–4, from top to bottom). In experiment C, most of the mice previously trained in a first touchscreen paradigm were assessed in a different task; because of their experience, early stages of pokey training were purposely skipped. For animals trained in stages with defined limited holding times (LHT), corresponding values are given in white. FD: Food Deprivation; IT: „Initial Touch“; MT: „Must Touch“; MI: „Must Initiate“; PI: „Punish Incorrect“; PT: „Pre-Training“.

16 male C57BL/6JRj mice were randomly assigned to 2 groups (n = 8 animals) and tested in one of the two versions of an “object-in-place” memory task involving the presentation of different (dPAL) *vs* similar (sPAL) stimuli during the main training phase (see figure 5).

Mice were food-deprived, then acclimated to the liquid reward in their home cage with 500 µL of condensed milk placed in a cup for 3 consecutive days. Afterwards, they were introduced in boxes with 250 µL of condensed milk into the magazine for a 20-min session of habituation. All mice had consumed the reward at the end of the session.

A pokey training procedure then started to train each animal to progressively detect and respond specifically to the window where a training stimulus appeared. In total, four different stages were included, namely “initial touch”, “must touch”, “must initiate” and “punish incorrect” stages. In all pokey training stages, only one training stimulus was displayed on the screen per trial, in one of the 3 possible windows. Training stimuli consisted of 40 possible various shapes that were pseudo randomly chosen.

In the “initial touch” paradigm, each trial started with the presentation of a training stimulus for a fixed duration (30 s) in one of the three possible locations of the screen. The end of this period coincided with the offset of the training stimulus and the delivery of the reward (8 µL) accompanied by the illumination of the magazine light and a tone. There was no inter-trial interval (ITI) at this stage: once the mouse had nose-poked into the food tray, a new trial started with the onset of a new training stimulus. Importantly, if the animal touched the training stimulus during its presentation, it received three times as much as the normal amount of reward (24 µL). Mice reached criterion when they were able to complete 36 trials in less than 60 min.

In the “must touch” paradigm, each trial started also with a training stimulus displayed in one of the three windows, but it remained visible until the mouse had nose-poked it. As previously, a successful nose-poke was followed by the illumination of the food tray, a tone and the delivery of the liquid reward (8 µL). An ITI (20 s) was introduced before the beginning of each new trial. When a mouse completed 36 trials in less than 60 min, the third stage of pokey training was started: the “must initiate” paradigm, during which the principle remained the same, except that animals had to nose-poke in the magazine before a training stimulus could be displayed on the screen. Same criteria as initial touch and must touch stages allowed to determine the start of the next stage.

In the “punish incorrect” paradigm, as before, a nose-poke of the training stimulus (correct response) was followed by the illumination of the food tray, a tone, and the delivery of the liquid reward (8 µL) with a 20 s ITI before a new trial could start. However, after a nose-poke of one of the two other blank windows (incorrect response), the training stimulus disappeared, the house light was turned on for a time-out period of 10 s and no reward was given. After 10 more seconds corresponding to the correction ITI, the mouse then had to complete a correction trial procedure. For that purpose, the last used training stimulus and its position were kept the same and were re-presented to the animal until it responded correctly. Importantly, correction trials were not counted in the total number of completed trials. Mice were directly brought to the next phase (dPAL or sPAL) when they achieved 36 trials in less than 60 min with an accuracy superior to 75% (minimum 27 correct responses) over two consecutive sessions.

**Figure 6:**
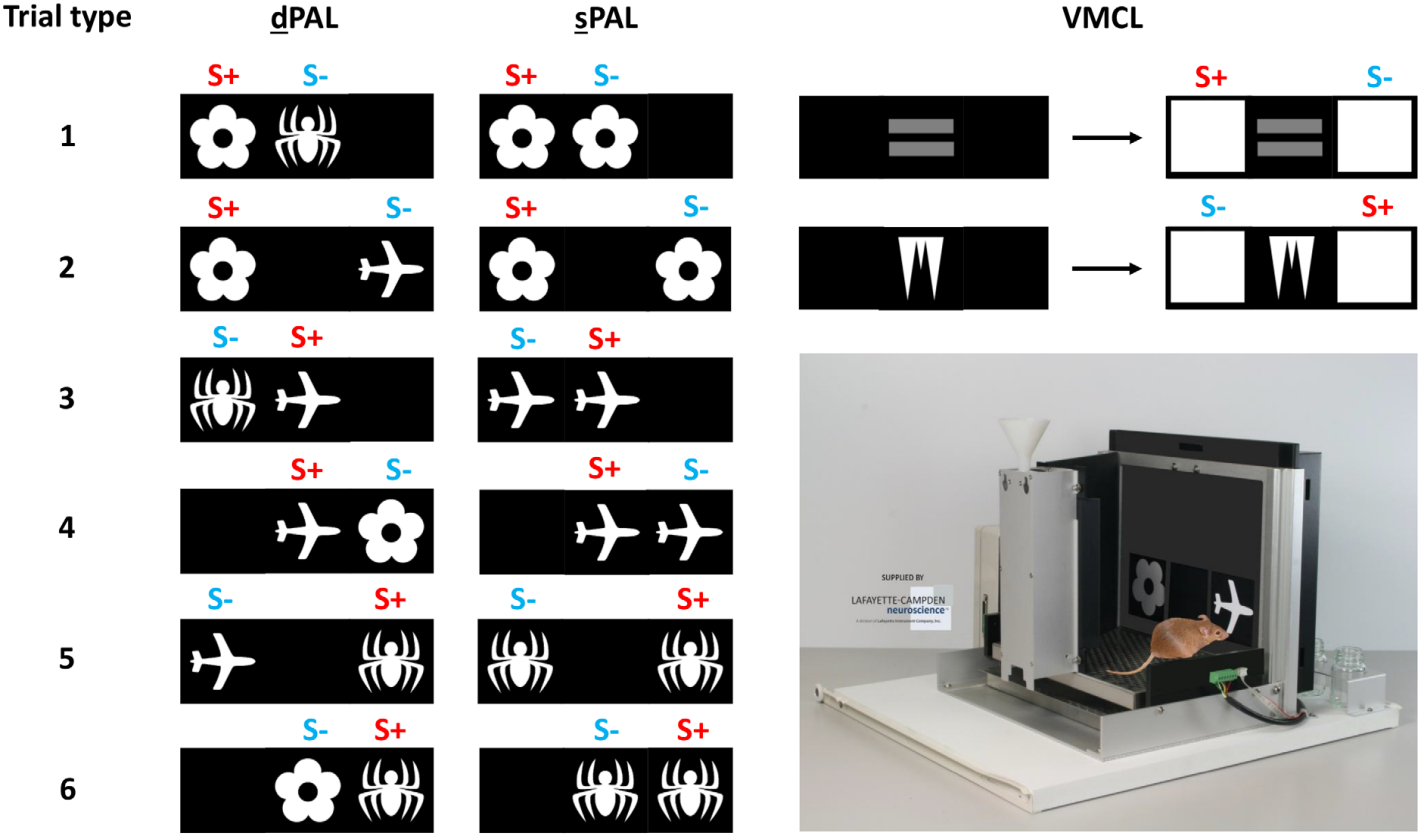
The different trial types in the two versions of the Paired-Associates Learning (dPAL and sPAL) tasks and in the Visuo-Motor Conditional Learning (VMCL) task. In both PAL paradigms, stimuli are rewarded when located in a specific location: left for the Flower, central for the Plane, right for the Spider. However, in a first variant of the task (dPAL), two different stimuli are presented at the same time, whereas two similar stimuli are presented in the second variant of the task (sPAL). In the VMCL paradigm, only two different trial types coexist for a given rule; note that the current rule can be inverted („If Equal appears, go Right; if Icicle appears, go Left“), which is why all groups were counterbalanced. S+: rewarded stimulus (correct response); S-: non-rewarded stimulus (incorrect response). Picture from Campden Instruments Ltd.; reprinted with permission.

In both variants of the PAL task, each mouse was required to learn specific paired-associations of stimuli and locations. Therefore, three discriminative stimuli (flower, plane, and spider) were used for a total of 6 possible trial types. Contrary to a previous paper [44], if the flower was also rewarded when presented in the left location, the plane was this time rewarded when presented in the central location, whereas the spider was rewarded when presented in the right location. Mice were recorded for a total of 50 sessions, with 36 trials per session. Each trial was initiated by nose-poking into the magazine. The tray light then switched off and a pair of stimuli appeared on the screen in 2 of the 3 possible locations: left, central, or right. These stimuli were different (dPAL) or similar (sPAL) ones; the latter condition was expected to be easier as animals did not have to discriminate between stimuli and locations within a same trial, but to discriminate between locations only (see figure 6). Among the 2 stimuli shown on the screen, one stimulus was the correct one (S+) and the other was the incorrect one (S−). When a mouse nose-poked the correct stimulus (case 1: correct response), both stimuli disappeared and the mouse was rewarded for a correct response as previously described. Entry to collect the reward turned off the tray light and started a 20 s ITI. Afterwards, the tray light was again illuminated and the mouse could nose-poke into the magazine to trigger the next trial by initiating the apparition of a new pair of stimuli on the screen. By contrast, if the mouse nose-poked the incorrect stimulus (case 2: incorrect response), the stimuli disappeared, the house light was turned on for a time-out period of 10 s and no reward was given. After 10 more seconds corresponding to the correction ITI, the mouse then had to complete a correction trial procedure. A correction trial consisted of the re-presentation of the last pair of stimuli in the same spatial configuration and was repeated until a correct response was given to the screen. As for the “punish incorrect” stage, correction trials were not counted in the total number of trials completed during the main training.

#### Experiment B: VMCL task in naive animals

32 male C57BL/6JRj mice were randomly divided into 4 groups (n = 8 animals) and trained under similar pokey training conditions before following specific “pretraining” and training programs. More precisely, in those two latter stages, animals had to nose-poke the stimuli in a limited holding time (LHT). Group 1 (no “pretraining”, VMCL with no LHT), group 2 (both “pretraining” and VMCL with no LHT), group 3 (“pretraining” LHT 10 s, VMCL LHT 5 s) and group 4 (“pretraining” LHT 10 s, VMCL LHT 3 s) thus aimed at defining the ideal conditions of learning (see figure 5).

As described above, food-deprivation, acclimation to the liquid reward and habituation to the environment also preceded the beginning of the testing procedure. All animals were trained in early pokey training stages (“initial touch”, “must touch” and “must initiate” stages) as for the PAL task with 3 differences: the nature of training stimuli, the locations where stimuli appeared over the different stages and finally the criterion. We used white squares as training stimuli. They appeared in one of the three possible locations during the initial touch stage, but only in one of the two lateral windows during “must touch” and “must initiate” stages. Finally, completion of each pokey training stage was achieved when mice performed 30 trials in less than 60 min.

Subsequent to this pokey training, groups 2 to 4 were given an additional “pretraining” stage to learn to nose-poke the touchscreen centrally, then laterally to get the reward. Before every new trial started, the mouse had to nose-poke into the magazine and exit the reward tray. A first white square then appeared in the central window, and remained until the animal nose-poked it. After the animal had touched the first stimulus, the stimulus disappeared and a second white square appeared in the left or right window of the screen. The position of this second stimulus was chosen pseudo randomly. The mouse then had to touch this second stimulus in a limited holding time (10 s for both groups 3 and 4) or not (group 2) to get the reward. If the mouse nose-poked the second stimulus before the fixed time limit was reached (case 1: correct trial), reward delivery was accompanied by illumination of the tray light and a tone as the stimulus disappeared. Entry to collect the condensed milk turned off the tray light and started the ITI. After the ITI period (20 s), the tray light was again illuminated, and a new trial could start. On the contrary, if the mouse did not nose-poke the second stimulus within the 10 s (case 2: omission, only for groups 3 and 4), the stimulus disappeared and no reward was given to the animal. Correction ITI period (10 s) followed a time out (10 s) during which the house light was illuminated. A correction trial procedure then started with the re-presentation of the first stimulus, followed by that of the second stimulus in the last proposed spatial configuration. Omissions were counted in the total number of trials. All groups were finally trained in the main task after groups 2, 3 and 4 reached the criterion of 30 trials completed in less than 60 min over 2 consecutive days (with less than 5 omissions per session for groups 3 and 4).

In the VMCL task, mice had to learn first to nose-poke the central window where a discriminative stimulus was displayed, then one of the 2 lateral locations depending on the nature of that central stimulus (see figure 6). They were recorded for a total of 30 sessions, with 30 trials per session. Each mouse first had to nose-poke into the magazine and exit the reward tray to initiate a trial. A first discriminative stimulus was then displayed in the central window, and remained until the animal nose-poked it. This discriminative stimulus was chosen pseudo randomly among 2 possible stimuli that were different in shapes and colors (white icicle *vs* grey equal). After the first central nose-poke, the initial stimulus remained visible and 2 white squares appeared laterally on the left and on the right of the screen. The mouse then had to touch one of these 2 stimuli to get the reward according to the predefined rule “If stimulus A appears, then go left; if stimulus B appears, then go right”, without (groups 1 and 2) or with a limited holding time (groups 3 and 4, respectively LTH = 5 and 3 s). Within each group trained in the VMCL task, the nature of visuo-motor associations was counterbalanced: half of the animals had to respond to the left panel when the grey equal was displayed and to the right panel when the white icicle was shown, whereas the other half had to respond to the right panel when the grey equal was displayed and to the left panel when the white icicle was shown (opposite rule).

If the mouse nose-poked the correct stimulus during the choice phase (case 1: correct trial), reward delivery was accompanied by illumination of the tray light and a tone. Entry to collect the condensed milk turned off the tray light and started the ITI. After the ITI period (20 s), the tray light was again illuminated and the mouse could initiate a new trial. If the mouse nose-poked the wrong stimulus during the choice phase (case 2: incorrect trial), all the stimuli disappeared, no reward was given to the animal and the house light was switched on for a 10 s time out period. After that, the house light was turned off again, and a correction ITI period (10 s) occurred before the tray light was switched on, after which a correction trial procedure occurred during which the same discriminative stimulus was presented first and the same lateral nose-poke was expected. Correction trials continued until the animal responded correctly to the screen. Finally, if the mouse didn’t manage to respond to the screen within the allocated time (case 3: omission in groups 3 and 4 only), the choice stimuli disappeared and no reward was given. A correction ITI period (10 s) followed a time out (10 s) during which the house light was illuminated. As for an incorrect trial, a correction trial procedure started. Importantly, and contrary to correction trials, omissions were counted in the total number of trials completed during the VMCL acquisition phase.

#### Experiment C: dPAL, sPAL and VMCL in animals previously assessed in touchscreen tasks

The end of experiments A and B coincided with the end of food restriction for all mice. Mice were then left in their cage with food and water *ad libitum* for 3 to 4 weeks. Afterwards, 32 out of the 48 male C57BL/6JRj mice that had been assessed in a first cognitive task were selected to acquire a new rule in the same touchscreen-equipped boxes (see figure 5): n = 8 mice tested in dPAL as task 1; n = 8 mice tested in sPAL as task 1; n = 8 mice tested in VMCL, conditions 2 as task 1; finally, n = 8 mice tested in VMCL, conditions 3 as task 1. We decided to use animals previously trained in conditions 2 and 3 in the VMCL task because those mice displayed really similar learning abilities. To our opinion, and contrary to conditions 1 (no “pretraining”) and 4 (different pattern of learning observed), those conditions allowed further comparisons in another touchscreen task.

Selected mice were again food-deprived and then maintained at 85% of their free-feeding weight. After two sessions of re-acclimation to the liquid reward in the home cage, the mice were returned to the operant chambers, starting directly at the “must initiate” stage due to their previous experience in touchscreens. All mice initially trained in variants of the PAL task were then trained in the VMCL task (conditions 2: both “pretraining” and VMCL with no LHT; counterbalanced stimuli as described in experiment B). By comparison, mice first trained in conditions 2 and 3 in the VMCL task started to acquire either the dPAL or the sPAL task (counterbalanced groups).

### Data Analysis

Five main parameters were explored: the accuracy (percentage of correct responses), the number of correction trials, correct/incorrect touch (time to nose-poke the correct/incorrect stimulus presented on the screen during the first presentation of stimuli on the screen) and magazine latencies (time to nose-poke into the magazine after giving the correct response on the screen). All collected data were expressed as means ± SEM. Accuracy data were plotted in blocks of 3 or 5 sessions depending on the nature of the task and therefore analyzed using a 2-way ANOVA with repeated measures on time, with a Bonferroni post-hoc analysis. By comparison, all other parameters, as global measures, were submitted to a 1-way ANOVA (VMCL task) with a Bonferroni post-hoc analysis or an unpaired t-test (sPAL or dPAL tasks) to detect any effect of the type of learning or conditions of testing.

Finally, to determine if there was a bias due to the use of discriminative locations in the touchscreen boxes, global measures corresponding to the repartition of correct responses among the 2 (VMCL task) or 6 (sPAL and dPAL tasks) possible trial types were generated from experiments A to C. These measures were analyzed with a 1-way ANOVA (sPAL or dPAL tasks) or with an unpaired t-test (VMCL task). All statistical analyses were performed with GraphPad Prism version 5.0 (GraphPad Software, San Diego, USA) and conducted with a significance level of p<0.05.
